# Me, Myself, and Not-I: Self-Discrepancy Type Predicts Avatar Creation Style

**DOI:** 10.3389/fpsyg.2020.01902

**Published:** 2021-01-15

**Authors:** Mitchell G. H. Loewen, Christopher T. Burris, Lennart E. Nacke

**Affiliations:** ^1^Department of Psychology, University of Waterloo, Waterloo, ON, Canada; ^2^HCI Games Group, The Games Institute, University of Waterloo, Waterloo, ON, Canada; ^3^Department of Psychology, St. Jerome's University, Waterloo, ON, Canada; ^4^Department of Communication Arts & Stratford School of Interaction Design and Business, University of Waterloo, Waterloo, ON, Canada

**Keywords:** avatars, self-discrepancy theory, values, video games, self-perception

## Abstract

In video games, identification with avatars—virtual entities or characters driven by human behavior—has been shown to serve many interpersonal and intraindividual functions (like social connection, self-expression, or identity exploration) but our understanding of the psychological variables that influence players' avatar choices remains incomplete. The study presented in this paper tested whether players' preferred style of avatar creation is linked to the magnitude of self-perceived discrepancies between who they are, who they aspire to be, and who they think they should be. One-hundred-and-twenty-five undergraduate gamers indicated their preferred avatar creation style and completed a values measure from three different perspectives: their actual, ideal, and ought selves. The average actual/ideal values discrepancy was greater among those who preferred idealized avatars vs. those who preferred realistic avatars. The average actual/ought values discrepancy was greater among those who preferred completely different avatars (i.e., fantasy/role-players) vs. those who preferred realistic avatars. These results, therefore, offer additional evidence that self-discrepancy theory is a useful framework for understanding avatar preferences.

## 1. Introduction

Over the past few decades, video games have become an integral component of popular culture and currently generate more revenue than the Hollywood movie industry (Nath, 2016). Unlike movies, many video games allow players to interact with and experience a digital environment via avatars. Although the use of the term “avatar” dates back to early multi-user dungeon text-based games, its application in virtual worlds and consequently video games designates control by a human actor (see Bailenson and Blascovich, 2004) rather than the artificial intelligence characteristic of game agents, often referring to digital non-player characters or entities, whose behavior is controlled through algorithms (Roth et al., 2017; Waltemate et al., 2018).

The first-person perspective provided by avatars facilitates a level of identification (Cohen, 2001) with the characters in the game that surpasses alternative visual media, such as film and television (Klimmt et al., 2009). Adopting a first-person perspective hinges on a sense of virtual embodiment, which is facilitated by an avatar's behavioral and photographic realism (Bailenson and Blascovich, 2004). The implications of such virtual embodiment or self-presence (Biocca, 1997; Jin and Park, 2009; Slater et al., 2009) are often considerable. For example, people will often conform to their digital self-representation even when that virtual body is unlike their own (cf. the so-called Proteus effect: Yee and Bailenson, 2007).

Consequently, avatars have been shown to serve a variety of psychological functions: interpersonal ones, such as social connection (e.g., Lomanowska and Guitton, 2014; Song and Fox, 2016) as well as intraindividual ones, such as self-expression (e.g., Sung et al., 2011) and identity exploration (e.g., Bessière et al., 2007; Hefner et al., 2007; Klimmt et al., 2010). The potential for positive applications of avatars in addressing domains, such as health and well-being (e.g., Fox and Bailenson, 2009; Jin, 2011; Behm-Morawitz, 2013) and the reduction of implicit racial bias (e.g., Banakou et al., 2016) have also been explored.

With the above as a backdrop, researchers have begun to identify factors that guide an individual's choices concerning how an avatar is chosen and/or customized, with a recurrent focus on the extent to which avatars resemble their users in physical and/or psychological characteristics. Mancini and Sibilla (2017, p. 275) recently stated that “there is at present no consensus on this issue, some studies have reported that players use their offline self as a starting point for the construction of their characters, and some others reported that players sometimes build characters which are totally disconnected from their offline self.”

In addition, the concept of self-presence helps frame the effect a player's virtual self can have on the “perception of one's body (i.e., body schema or body image), physiological states, emotional states, perceived traits, and identity” (Biocca, 1997). This led to research suggesting that an avatar could have a positive influence on well-being and health appearance and behaviors (Fox and Bailenson, 2009; Jin, 2011; Behm-Morawitz, 2013). Along those lines, studies investigated the concept of parasocial interaction with an avatar, where self-presence was understood as “the extent of game players' interpersonal involvement with their avatar and the extent to which game players perceive themselves as interacting with the avatar” (Jin and Park, 2009).

Paralleling Mancini and Sibilla (2017) and other researchers (e.g., Dunn and Guadagno, 2012; Villani et al., 2016), we suggest that self-discrepancy theory (SDT) (Higgins, 1987) provides a useful framework for making sense of avatar choices. According to SDT, the self can be understood in terms of three domains:

1. the *actual self* (the attributes that someone—self or other—perceives that the target person actually possesses);
2. the *ideal self* (the attributes that someone wants the target person to possess), and
3. the *ought self* (the attributes that someone believes that the target person should possess).

Discrepancies between the actual self and either self-guide (i.e., the ideal self or the ought self) have been posited to evoke distinct classes of emotions as well as motivation to resolve the perceived discrepancies.

As Mancini and Sibilla (2017) themselves noted, extant avatar research inspired by SDT has focused on the actual and ideal selves; the ought self has been overlooked. We posit that discrepancies centered on the ought self may help explain why some individuals prefer avatars that are “totally disconnected from their offline self” (p. 275), however. Testing this possibility was a primary goal of the present research.

Our methodology incorporated an operationalization of Neustaedter and Fedorovskaya (2009)'s avatar preference typology. Within their framework, so-called “Realistics” aim for continuity between their digital and real-life selves by attempting to make the former similar to the latter in terms of appearance. “Ideals” are more selective, as their constructed avatars reflect the “best” parts of themselves, and/or traits and characteristics to which they aspire. In contrast, “Fantasies” and “Roleplayers” maintain a clear distinction between their real and virtual selves, which often differ markedly. Indeed, an adopted avatar allows the player an opportunity to explore virtual worlds through the eyes of a persona quite unlike themselves: Fantasies do so via one's virtual self, whereas Roleplayers do so via multiple virtual selves. Overall, then, the differences between chosen avatars and their player-creators range from minimal (Realistic) to moderate (Ideal) to substantial (Fantasy/Roleplayer).

In SDT terms, we would expect Ideals to be more likely to perceive a discrepancy between their actual and ideal selves compared to Realistics: Ideals' avatars reflect the “best” parts of players and/or traits and characteristics to which they aspire, whereas the avatars of Realistics are arguably less aspirational, instead closely resembling the players themselves. Like those of Ideals, Fantasies/Roleplayers' virtual selves differ from their real-world personas, but the differences are so great that they are not likely to be the result of simple aspiration. Consequently, we would have no clear conceptual basis for predicting that the average actual-ideal self-discrepancy of Fantasies/Roleplayers would differ from those of Realistics.

We would, however, expect Fantasies/Roleplayers to be more likely to perceive actual-ought self-discrepancies relative to Realistics. Actual-ought self-discrepancies have been linked conceptually to resentment and fear of negative social evaluation (Higgins, 1987). Consequently, identifying with an avatar that is wholly different from the real-world self-amidst the protective anonymity of virtual environments may allow Fantasies/Roleplayers dealing with actual-ought self-discrepancies to explore and express personal attributes perceived to be too taboo or risky to own or express in the real world (Crenshaw and Nardi, 2014; Mancini and Sibilla, 2017). In contrast, we would have no conceptual basis for expecting Ideals and Realistics to differ with respect to actual-ought self-discrepancies.

To test these hypotheses, participants who had created or customized at least one avatar as part of an online gaming experience completed a brief, cross-culturally validated values measure three times—that is, from the perspective of their actual, ideal, and ought selves (meaning: completion of a short personality inventory from the perspective of the actual, ideal, and avatar selves in Mancini and Sibilla, 2017). They also indicated their preferred style of avatar creation based on descriptions adapted from Neustaedter and Fedorovskaya (2009). We subsequently computed actual-ideal and actual-ought values discrepancy scores and compared the resulting means among three groups: Realistics, Ideals, and Fantasies/Roleplayers (henceforth referred to as “Differents”)^1^.

## 2. Method

In this section, all dependent measures, conditions, and data exclusions are reported. The sample size was maximized in the context of practical and temporal constraints—specifically, the number of study-specific volunteer slots allotted by the research pool coordinator coupled with the first author's fixed timeframe for completing his thesis on which this report is based. The study received ethics approval from the ethics board at the authors' home institution.

### 2.1. Participants

One hundred fifty-two undergraduates registered with the psychology research pool at the University of Waterloo agreed to participate in an online study described as investigating “the relationship between video game players and their in-game avatars to better understand how and why players create the avatars that they do” in exchange for extra course credit. Would-be participants were asked to sign up for the study only if they had previously played a Massively Multiplayer Online Game (MMOG) that involved avatar creation. Median age was 20, with 97% of the sample between ages 17 and 24.

Twenty-seven participants were excluded from the final data set. Specifically, based on their responses to the screening questions, three had not played a game that involved avatar customization and seven did not provide a name and/or description of the game they had played. In addition, 13 participants reported nearly identical responses (e.g., “6”) to every question across all selves and avatars (suggesting inattention to item content), one did not respond to the avatar-creation-style item, one did not complete the ought-self measure, one completed the study twice (and so the second set of responses was removed), and one did not complete any of the key measures. Thus, the final sample consisted of 125 participants (51 female, 72 male, 2 other/no response; 38% Euro-Canadian, 38% East Asian, 24% other).

### 2.2. Materials

#### 2.2.1. Screening Questions

To ensure that participants met inclusion criteria (see above), they were asked to specify which MMOG they had played the most, to provide a brief description of the game, and to describe if the game allowed for avatar customization (the three MMOGs most frequently listed by final-sample participants were *MapleStory* [*n* = 19], *World of Warcraft* [*n* = 19], and *RuneScape* [*n* = 14]; no other listed game exceeded *n* = 5). They were also asked how long (in months) they had played the specified game (*M* = 18.24; *SD* = 21.28).

#### 2.2.2. Self-Discrepancy Measure

To assess the magnitude of participants' actual/ideal and actual/ought self-discrepancies across a broad, significant personal domain, participants were asked to complete the short version of Schwartz's Value Survey (SSVS) as presented in Lindeman and Verkasalo (2005) (see also Appendix) under three different instructional sets using a −2 to 14 response range with the following anchors: −2 = opposed to my values; 0 = not important; 6 = important; 12 = very important; 14 = of supreme importance. Thus, participants rated the importance of values, such as “power” and “self-direction” from the perspective of: (1) the actual self (i.e., “how you truly see yourself”); (2) the ideal self (i.e., “how you would like to be”); and (3) the ought self (i.e., “how you think others think you should be”).

Mean actual-ideal and actual-ought discrepancy scores for each individual were computed by averaging the absolute values of the discrepancy scores for each of the 10 relevant values pairs (e.g., *actual Hedonism—ideal Hedonism*, or *actual Benevolence—ought Benevolence*). Given this computational strategy and the fact that the SSVS uses single items to assess each of the 10 values represented within Schwartz's circumplex model, an internal consistency coefficient could not be computed (but see Lindeman and Verkasalo, 2005 for psychometric information concerning the SSVS in its original form).

#### 2.2.3. Avatar Creation Style

Participants selected their preferred avatar creation style from three descriptions based on Neustaedter and Fedorovskaya (2009)—that is, *Realistic, Ideal*, and *Different*, respectively:

**When you create avatars in games, which of the following statements best describes you (choose only one)?**

1. When I create avatars in games, I tend to create them as realistic and similar to myself as possible.
2. When I create avatars in games, I tend to create them as an idealized version of myself.
3. When I create avatars in games, I tend to create them as someone distinctly different from myself.

#### 2.2.4. Procedure

Participants completed the study online in a time and location of their choosing. They first completed the screening questions followed by the three versions of the SSVS (in fixed actual/ideal/ought order) and then indicated their preferred avatar creation style. They subsequently provided basic demographic information, reported how many hours per week they spent playing video games (*M* = 9.62; *SD* = 10.35)^2^, and received online debriefing^3^.

## 3. Results

The avatar creation style breakdown in the present sample was 27 Realistics, 68 Ideals, and 30 Differents. Preliminary two-way analyses of variance (ANOVAs) revealed that neither the gender main effect nor the gender × avatar creation style interaction was significant for either the actual-ideal or the actual-ought self-discrepancy scores (all *p*_*s*_ > 0.10), so gender will not be discussed further^4^. Thus, for hypothesis testing purposes, we conducted separate one-way analyses of variance (ANOVAs) to test for possible links between avatar creation style and actual-ideal and actual-ought self-discrepancies. Given our focus on composite scales rather than single Likert-type items, and given that homogeneity of variance tests yielded non-significant results for both the actual-ideal (*p* = 0.371) and actual-ought discrepancy scores (*p* = 0.441), use of the F-statistic is defensible (see also Carifio and Perla, 2008).

**Figure 1:**
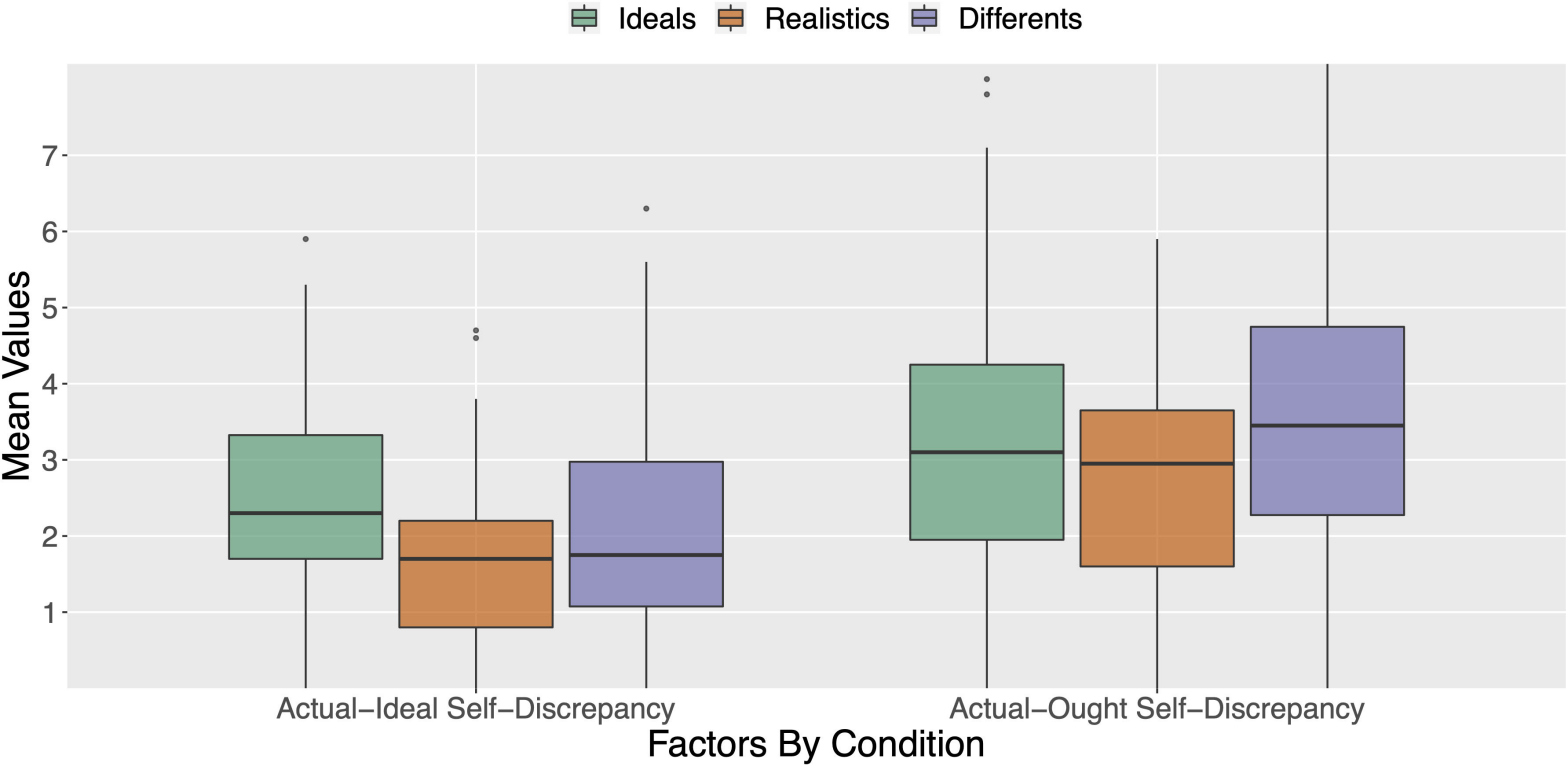
Comparison of Realistics, Ideals, and Differents for their actual-ideal and actual-ought self-discrepancy scores.

There was a significant main effect of avatar creation style on actual-ideal self-discrepancy scores, $F_{\left(2,122\right)}=3.36,p=0.038,\eta_{p}^{2}=0.052$. *Post-hoc* pairwise comparisons (least significant difference procedure) revealed the expected pattern (see Figure 1): Ideals reported significantly (*p* = 0.011) higher actual-ideal self-discrepancy scores (*M* = 2.57; *SD* = 1.81) compared to Realistics (*M* = 1.81; *SD* = 1.02). In contrast, neither Ideals nor Realistics differed significantly from Differents (*M* = 2.42; *SD* = 1.37; *p* = 0.590 and *p* = 0.080, respectively).

There was also a significant main effect of avatar creation style on actual-ought self-discrepancy scores, $F_{\left(2,122\right)}=3.15,p=0.046,\eta_{p}^{2}=0.049$. *Post-hoc* pairwise comparisons revealed the expected pattern: Differents reported significantly (*p* = 0.047) higher actual-ought self-discrepancy scores (*M* = 4.30; *SD* = 2.18) compared to Realistics (*M* = 3.31; *SD* = 1.85). Actual-ought self-discrepancy scores were also significantly (*p* = 0.018) higher for Differents than for Ideals (*M* = 3.32; *SD* = 1.72). In contrast, actual-ought self-discrepancy scores for Realistics and Ideals did not differ significantly (*p* = 0.972).

## 4. Discussion

Guided by self-discrepancy theory (SDT), we conducted the present research to gain a better understanding of how and why video game players select or create the avatars that they do. We reasoned that players' preferred style of avatar creation could be linked to the magnitude of self-perceived discrepancies between who they are, who they aspire to be, and who they think they should be. To test this idea, MMOG players indicate their preferred avatar creation style and completed a values measure from three different perspectives. Methodologically speaking, our approach differed from previous discrepancy-based avatar research in at least three ways. First, our participants completed all three discrepancy-related measures from a self-perspective (rather than one or more from the perspective of an avatar). Second, an ought self-measure was included among these three. Third, our computational approach focused solely on the magnitude of self-discrepancies, not their direction (vs., e.g., Mancini and Sibilla, 2017).

As hypothesized, the perceived values discrepancy between actual and ideal self averaged higher among those who preferred idealized avatars compared to those who preferred realistic avatars; those who preferred completely different avatars (i.e., fantasy/role-players) averaged in between. Also as hypothesized, the perceived values discrepancy between actual and ought self averaged higher among those who preferred different avatars compared to those who preferred realistic avatars; the actual-ought discrepancy among fantasy/role-players (i.e., different avatars) also averaged higher compared to those who preferred idealized avatars.

These results contribute to the existing empirical literature concerning the extent to which avatars serve a compensatory function among those who perceive gaps between who they are and who they want to be or (think they) should be. That is, whereas idealized avatars embody aspirations, wholly different avatars seem to reflect a casting off of perceived demands within the relative safety of a virtual game world's “Magic Circle” (where players will conform to how they represent themselves digitally, manifesting in deviant or aspirational behavior in line with the Proteus effect as discussed by Yee and Bailenson, 2007). In contrast, true-to-self (realistic) avatars tend to be preferred by those who perceive their various selves to be in comparative alignment.

Understanding avatar creation style from an SDT perspective that includes the ought self as well as the ideal self opens up intriguing avenues for subsequent research. Indeed, in its original formulation, SDT was intended to help understand emotions, with actual-ideal discrepancy mapping onto depressive affect and actual-ought discrepancy mapping onto anxiety (Higgins, 1987). It might therefore be worthwhile to explore the potential for avatar creation style to serve not only as a proxy indicator of psychological well-being, but also as a clue concerning the domain(s) in which adjustment difficulties may lie. For example, a strong preference for “different” avatars might suggest that an individual is struggling with one or more identity elements in their real life (e.g., sexual orientation, religious disillusionment) that may be subject to censure in their social environment. Extending this reasoning, shifts in preferred avatar creation style over time could be of diagnostic value.

The psychosocial consequences of discrepancy-congruent or discrepancy-incongruent gameplay should also be explored. For example, do individuals with a substantial actual-ideal discrepancy feel better after playing as an idealized avatar? Would individuals who lack substantive self-discrepancies feel disoriented after playing as a fantasy/role-play avatar? How enduring are such effects?

The adoption of specific avatar creation styles could have therapeutic value. Thus, in line with research suggesting positive effects of feeling self-present in a game world (Fox and Bailenson, 2009; Jin, 2011; Behm-Morawitz, 2013), game developers could actively promote diverse avatar creation styles based on the needs of their community. Given the volatility of online communities, this could be a helpful tool for game community managers seeking to improve the collective well-being and/or possibly reduce the toxicity of online gaming communities. In this sense, knowledge of avatar creation styles provides a missing link between the self-expressive world of virtual characters and the real-life interaction displayed in out-of-game community behaviors.

With respect to limitations, the present study sampled only Canadian psychology undergraduates, but gender and ethnic diversity was considerable (40% women, 62% non-Euro-Canadian). The avatar creation style instrument created for this study relied on a single-item, forced-choice format, which may have sacrificed some information that multi-item, continuous measures could have provided. For example, a basic distinction between an avatar's appearance and the avatar's in-game behavior could prove important. Moreover, although avatar style preferences are at least somewhat stable within individual players (see Mancini and Sibilla, 2017), contextual factors can also shape specific choices (e.g., Triberti et al., 2017). Discrepancy scores in the present study were generated based on a novel administration of a values scale, although the values dimensions assessed have been among the most comprehensive and cross-culturally validated constructs in psychology (see Lindeman and Verkasalo, 2005).

Notwithstanding the present study's limitations, our results demonstrate SDT's usefulness with respect to understanding the link between players and their avatars. Indeed, our results suggest that game designers would do well to ensure that players have the tools to fashion avatars that feel “right,” for avatar creation appears to be driven—at least in part—by the oughts and ideals that the players carry within them.

## Data Availability Statement

The raw data supporting the conclusions of this manuscript will be made available by the authors, without undue reservation, to any qualified researcher.

## Ethics Statement

The studies involving human participants were reviewed and approved by Psychology Delegated Ethics Review Committee (DERC), ORE #: 21832. The patients/participants provided their electronic informed consent to participate in this study.

## Author Contributions

ML designed the studies, performed the statistical analysis together with CB, interpreted the study results together with CB and LN, and wrote the first full draft of the manuscript together with CB. LN co-supervised ML, edited the drafts, and provided the feedback on the project and on the manuscript as well as rewriting portions of it from the last draft to this submission. All authors contributed to the article and approved the submitted version.

## Conflict of Interest

The authors declare that the research was conducted in the absence of any commercial or financial relationships that could be construed as a potential conflict of interest.

## Footnotes

^1^Neustaedter and Fedorovskaya (2009) suggested that Roleplayers and Fantasies might constitute conceptually distinct subtypes of “Different” avatar creators. Of the 30 Differents in our sample, only six indicated that they created multiple avatars (which is the primary distinguishing feature of Roleplayers). Consequently, we did not attempt analyses intended to differentiate the two Different subtypes in the present study.

^2^One-way analyses of variance (ANOVAs) revealed no significant relationship between avatar creation style and either months of self-reported experience with the specified MMOG or self-reported hours of video game play per week (both *Fs* < 1), so neither time variable will be discussed further.

^3^As part of a secondary research question, participants also completed a 10-item measure, based on Livingston et al. (2014), of the economic (utilitarian) values that players ascribe to their avatars. The measure was completed once for each avatar participants indicated having created or customized. Results associated with this measure will not be discussed here.

^4^For more poignant discussions of constraints on gender expression in MMOGs, see McArthur et al. (2015), McArthur (2017), and Pace et al. (2009).

## Appendix

### Short Schwartz's Value Survey

In the following questionnaire, you will be asked about the importance of a range of values from the perspective of your actual, ideal, and ought selves.

Your **“actual self”** refers to **how you truly see yourself**. Your **“ideal self”** refers to **how you would like to be**. Your **“ought self”** refers to **how you think others think you should be**.

Each of the next three pages of the questionnaire will focus on one of these selves.

1. Please rate the importance of the following values as guiding principles from the perspective of how you truly see yourself (that is, your “actual self”).
2. Now rate the importance of the same values from the perspective of how you would like to be (that is, your “ideal self”).
3. Finally, please rate these values once more from the perspective of how others think you should be (that is, your “ought self”).

(−2 = Opposed to my values, 0 = Not Important, 6 = Important, 12 = Very Important, 14 = Of Supreme Importance) POWER (social power, authority, wealth)

ACHIEVEMENT (success, capability, ambition, influence on people and events)

HEDONISM (gratification of desires, enjoyment in life, self-indulgence)

STIMULATION (daring, a varied and challenging life, an exciting life)

SELF-DIRECTION (creativity, freedom, curiosity, independence, choosing one's own goals)

UNIVERSALISM (broad-mindedness, beauty of nature and arts, social justice, a world at peace, equality, wisdom, unity with nature, environmental protection)

BENEVOLENCE (helpfulness, honesty, forgiveness, loyalty, responsibility)

TRADITION (respect for tradition, humbleness, accepting one's portion in life, devotion, modesty)

CONFORMITY (obedience, honoring parents and elders, self-discipline, politeness)

SECURITY (national security, family security, social order, cleanliness, reciprocation of favors).
